# Real‐time indocyanine green perfusion assessment demonstrates regional vascularity and loss of perfusion in the anterior cruciate ligament during knee arthroscopy

**DOI:** 10.1002/jeo2.70758

**Published:** 2026-05-22

**Authors:** Yasutoshi Ikeda, Kazushi Horita, Kodai Hamaoka, Shinichiro Okimura, Yohei Okada, Tomoaki Kamiya, Makoto Emori, Atsushi Teramoto

**Affiliations:** ^1^ Department of Orthopaedic Surgery Sapporo Medical University School of Medicine Sapporo Japan

**Keywords:** anterior cruciate ligament, indocyanine green, perfusion assessment, tissue vascularity

## Abstract

**Purpose:**

To evaluate the feasibility of real‐time intra‐articular indocyanine green (ICG) fluorescence imaging for visualising anterior cruciate ligament (ACL) vascularity during knee arthroscopy and to explore perfusion patterns associated with ligament integrity.

**Methods:**

In this prospective exploratory study, patients undergoing knee arthroscopy with informed consent were consecutively enrolled. ICG perfusion assessment was performed without tourniquet use using near‐infrared arthroscopic imaging. A single intravenous bolus of ICG was administered, and fluorescence dynamics were recorded. In primary ACL rupture cases, fluorescence at the torn stump was assessed qualitatively as present or absent. In ACL‐intact knees without advanced osteoarthritis, quantitative region‐of‐interest–based analysis was performed to compare medial and lateral perfusion within the ligament. Paired comparisons were conducted using the Wilcoxon signed‐rank test.

**Results:**

Thirty‐two knees underwent ICG perfusion assessment. Fluorescence was successfully visualised in all cases, and no ICG‐related complications were observed. All primary ACL rupture cases demonstrated absence of fluorescence at the torn stump, indicating loss of local perfusion. In ACL‐intact knees (*n* = 10), quantitative analysis showed consistently higher fluorescence intensity in the medial portion compared with the lateral portion (*p* < 0.001). These Quantitative findings should be interpreted as exploratory.

**Conclusion:**

Real‐time intra‐articular ICG fluorescence imaging is feasible during routine knee arthroscopy and enables visualisation of ACL vascularity. Distinct perfusion patterns associated with ligament integrity were observed in this exploratory cohort. These findings provide preliminary biological insight into intra‐articular microcirculation and establish a foundation for future outcome‐oriented studies.

**Level of Evidence:**

Level IV.

**Abbreviations**
ACL: anterior cruciate ligament
AL: anterolateral
ICC: intraclass correlation coefficient
ICG: indocyanine green
M/L ratio: medial‐to‐lateral fluorescence intensity ratio
ROI: region‐of‐interest

## INTRODUCTION

Advances in arthroscopic techniques have markedly improved clinical outcomes following anterior cruciate ligament (ACL) reconstruction. Nevertheless, graft failure and re‐tear rates remain unacceptably high—reported at 2.6%–29.5% after ACL reconstruction [9, 25, 28, 30]. Despite decades of technical refinement, no intraoperative method currently allows surgeons to directly assess the biological healing potential of intra‐articular soft tissues, a factor fundamental to postoperative success.

**Figure 1:**
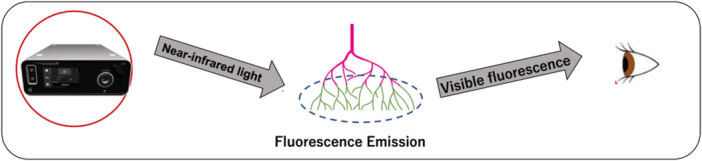
Near‐infrared fluorescence excitation and visible fluorescence. Schematic illustration of near‐infrared fluorescence imaging using an arthroscopic system. Near‐infrared excitation light illuminates the tissue, and the emitted fluorescence is captured to visualise tissue perfusion.

Indocyanine green (ICG) fluorescence imaging (Figure 1) has become an established modality for real‐time perfusion assessment in gastrointestinal, plastic and urologic surgery [1, 11, 15, 16, 19, 20, 22], where it has significantly reduced complications such as anastomotic leakage and reoperation rates [3, 32]. However, its application in orthopaedic arthroscopy remains limited, particularly in the knee. Since Watanabe introduced knee arthroscopy in 1957 [34], technological innovations have enhanced visualisation and therapeutic capability; nevertheless, no technique has enabled direct real‐time evaluation of intra‐articular microcirculation.

Recent advances in arthroscopic optics and near‐infrared sensitivity have made intra‐articular fluorescence imaging technically feasible under routine arthroscopic conditions [23]. This development creates an opportunity to extend arthroscopic evaluation beyond morphology toward biological assessment, particularly in procedures where vascularity and tissue viability influence healing potential [4, 8, 33].

Therefore, the purpose of this exploratory study was to determine whether real‐time ICG perfusion assessment during routine knee arthroscopy provides biologically meaningful information regarding ACL tissue viability. It was hypothesised that intact ACL tissue would demonstrate preserved and region‐specific perfusion patterns in vivo, whereas disrupted or degenerated tissue would show reduced or heterogeneous perfusion. By characterising intraoperative ACL perfusion patterns, this study aimed to demonstrate that intra‐articular ICG perfusion assessment can be performed safely during routine arthroscopy and to generate preliminary biological insight into ACL vascularity.

## METHODS

This prospective clinical study was approved by the Institutional Review Board of Sapporo Medical University (approval date: 23 May 2025; approval number: 170–20). During the study period, ICG perfusion assessment was performed in a consecutive series of patients undergoing knee arthroscopy who provided written informed consent for study participation. All enrolled patients underwent ICG perfusion assessment as part of the exploratory feasibility protocol; subgroup analyses were subsequently performed only in cases that met predefined ACL‐status criteria.

**Figure 2:**
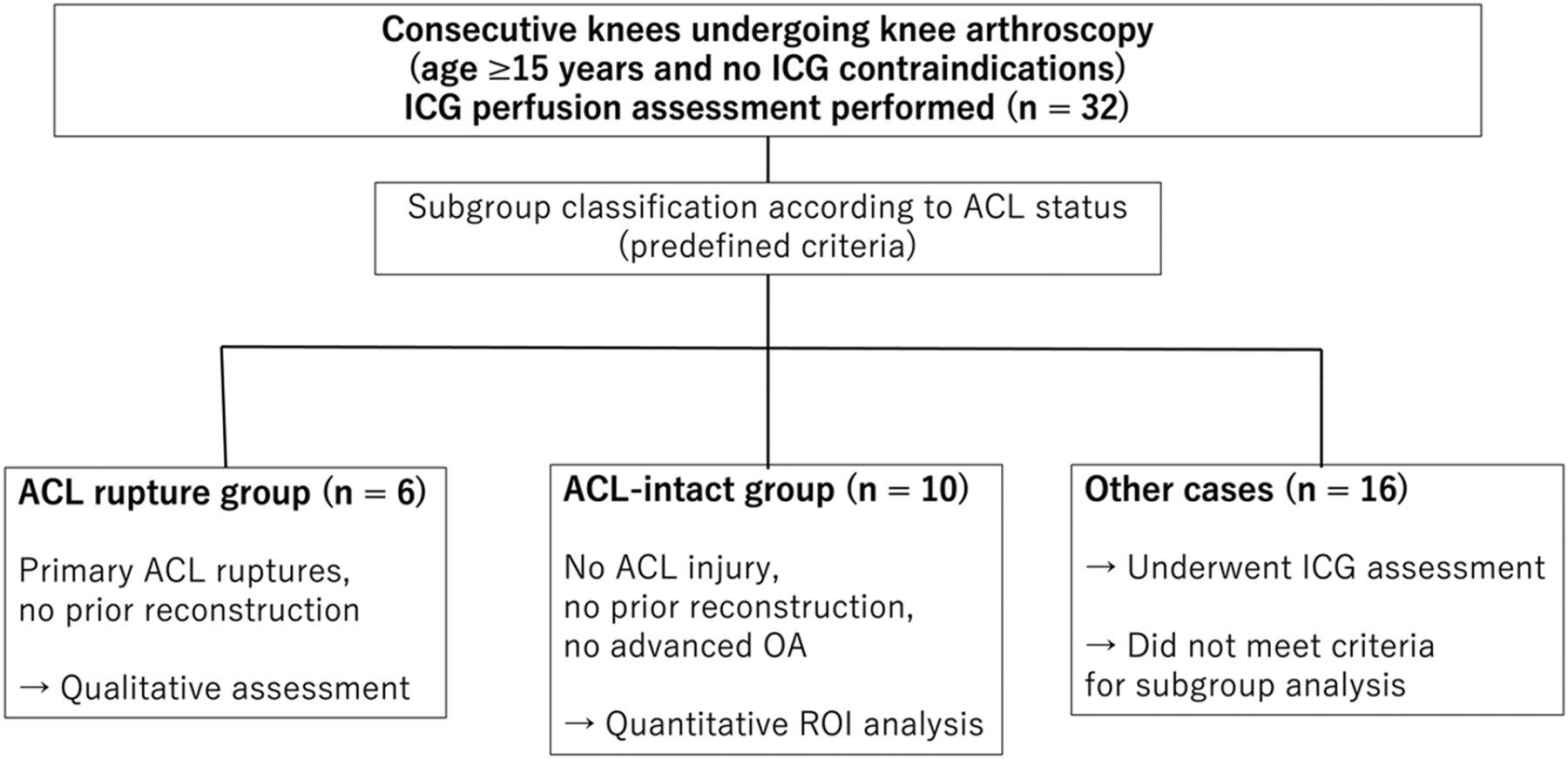
Flowchart illustrating patient selection and subgroup allocation. All 32 knees underwent indocyanine green (ICG) perfusion assessment during arthroscopy as part of the exploratory feasibility protocol. Subgroup analyses were performed according to predefined anterior cruciate ligament (ACL)‐status criteria: 6 knees with primary ACL rupture underwent qualitative assessment of perfusion at the torn stump, and 10 ACL‐intact knees underwent quantitative region‐of‐interest analysis. The remaining 16 knees did not meet criteria for either subgroup analysis and were therefore not included in specific analyses.

Inclusion criteria consisted of consecutively enrolled patients who underwent knee arthroscopy between 3 June 2025 and 9 December 2025 and consented to participate in the study. Patients younger than 15 years or with known contraindications to ICG were excluded. Contraindications to ICG administration included multiple allergies, iodine or indocyanine green hypersensitivity, severe hepatic dysfunction, or other conditions deemed inappropriate by the attending anaesthesiologist. All enrolled patients underwent routine preoperative blood testing, and none had evidence of hepatic dysfunction, including abnormal liver enzyme levels (e.g., aspartate aminotransferase or alanine aminotransferase), at the time of surgery. The patient selection process, including inclusion and exclusion criteria and subgroup allocation, is summarised in Figure 2.

Two senior surgeons performed all procedures. General anaesthesia with peripheral nerve block was administered, and patients were positioned supine. No tourniquet was applied during ICG perfusion assessment. Arthroscopy was performed using the 1788 Platform (Stryker Japan). Standard anteromedial and anterolateral (AL) portals were used, with far anteromedial or far anterolateral portals added when necessary. The irrigation pressure was maintained at 30 mmHg using a pump system with Arthromatic irrigation fluid (Baxter Japan).

ICG (Diagnogreen for Injection, 25 mg; Daiichi Sankyo) was prepared according to Kamimura et al. [13]. ICG (25 mg) was dissolved in 10 mL of distilled water. The anesthesiologist administered a 2‐mL intravenous bolus (2.5 mg/mL), followed by a 20‐mL saline flush. Patients were prospectively monitored for adverse events related to ICG administration, including allergic reactions and hemodynamic instability. Safety outcomes were recorded based on anaesthetic records, intraoperative observations and postoperative clinical assessments during hospitalisation.

**Figure 3:**
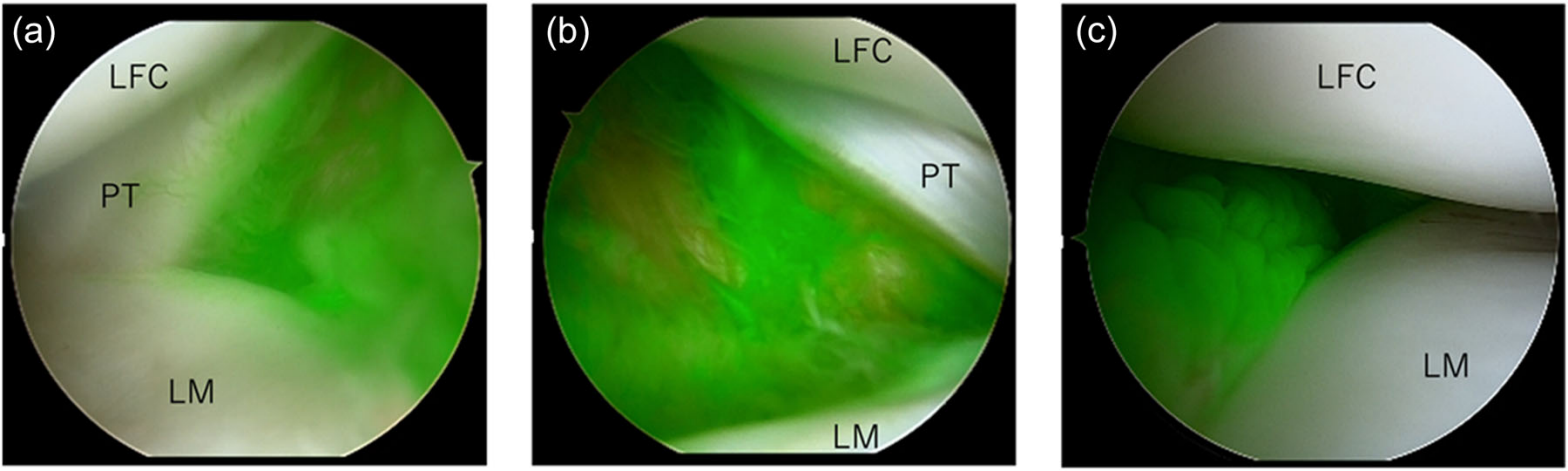
Fluorescence of the synovium anterior to the popliteus tendon. Representative arthroscopic images demonstrating indocyanine green (ICG) fluorescence of the synovium anterior to the popliteus tendon observed from the anterolateral portal. The fluorescence onset time and duration were measured at this standardised location. The lateral femoral condyle, popliteus tendon and lateral meniscus are shown as anatomical landmarks. (a) 58‐year‐old male, left knee. (b) 44‐year‐old male, right knee. (c) 20‐year‐old male, right knee.

Fluorescence was observed from the AL portal, with a focus on the synovium anterior to the popliteus tendon sulcus (Figure 3). Two temporal variables were recorded: time to fluorescence onset (flush to first detectable signal) and duration of fluorescence. Representative real‐time fluorescence dynamics of the synovium anterior to the popliteus tendon are shown in Video S1.

The initial consecutive cohort included patients undergoing arthroscopic procedures for various indications, including meniscal repair, ACL reconstruction, revision ACL reconstruction, high tibial osteotomy, hardware removal procedures, posterior cruciate ligament reconstruction and medial patellofemoral ligament reconstruction.

For the purpose of perfusion analysis, knees without ACL injury or prior ACL reconstruction were categorised as ACL‐intact. These cases included procedures not directly related to the ACL (e.g., meniscal repair, HTO, hardware removal procedures and MPFL reconstruction). Because the primary analysis relied on within‐knee comparisons of fluorescence intensity rather than comparisons between different patients or surgical indications, the potential confounding effects related to heterogeneous surgical indications were considered limited.

### Subgroup classification according to ACL status

For analysis purposes, cases were categorised according to ACL status. ACL rupture cases (*n* = 6) were defined as knees with primary ACL rupture without prior reconstruction diagnosed based on clinical and imaging findings. The mean interval from injury to surgery was 86.9 days (range, 20–210 days). ACL‐intact knees (*n* = 10) were defined as knees without ACL injury, history of ACL reconstruction, or advanced osteoarthritis and were included in the quantitative ROI‐based analysis. The remaining cases did not meet criteria for either subgroup analysis.

### Assessment in ACL rupture cases

For primary ACL ruptures, fluorescence at the torn stump was assessed qualitatively as either present or absent.

### ROI‐based quantitative analysis in ACL‐intact knees

Quantitative analysis was performed in knees without ACL injury, history of ACL reconstruction, or advanced osteoarthritis (ACL‐intact group, *n* = 10). For each knee, six regions of interest (ROIs) were manually defined on the ACL substance—three on the medial aspect and three on the lateral aspect. Medial ROIs were placed within the medial one‐third of the ACL substance, and lateral ROIs within the lateral one‐third to standardise regional sampling across knees.

Peak‐frame fluorescence intensity was assessed using standardised still images captured at the time of maximal ICG enhancement. During perfusion assessment, the knee was positioned at approximately 60° of flexion in a gravity‐assisted dependent position. Because some procedures were performed with a leg holder whereas others were performed without one, this mid‐flexion dependent position was selected to standardise imaging conditions and ensure consistent and reproducible evaluation of ACL perfusion.

Image processing was performed using Fiji (ImageJ, National Institutes of Health, USA). Images were separated into RGB channels, and analysis was conducted exclusively on the green channel. Fluorescence intensity values were extracted from the processed images for all defined ROIs.

Because absolute fluorescence intensity in arthroscopic imaging can be influenced by acquisition‐related factors, quantitative interpretation focused on relative regional differences within the same knee rather than absolute signal magnitude. Accordingly, a medial‐to‐lateral fluorescence intensity ratio (M/L ratio) was calculated for each knee by dividing the mean medial ROI intensity by the mean lateral ROI intensity. This ratio‐based approach was used as the primary metric for regional perfusion comparison.

### Statistical analysis

For quantitative analysis, fluorescence intensities from the three medial and three lateral ROIs were averaged within each knee to generate per‐knee medial and lateral values. Because medial and lateral measurements were obtained from the same knee, paired comparisons were performed.

Normality of paired medial–lateral fluorescence intensity differences was assessed using the Shapiro–Wilk test. Given deviation from normality and the small sample size (*n* = 10), a non‐parametric Wilcoxon signed‐rank test was selected.

A simulation‐based a priori power estimation for the paired analysis indicated that seven knees would provide more than 80% power to detect the observed effect size; therefore, the analysed sample of 10 ACL‐intact knees was considered sufficient.

As a secondary sensitivity analysis, ROI‐level fluorescence intensities were compared between medial and lateral regions using the Mann–Whitney *U* test. However, because multiple ROIs were sampled within each knee, the per‐knee paired comparison was considered the primary statistical inference.

Effect size for the paired comparison was calculated using Cliff's delta. A two‐tailed *p*‐value < 0.05 was considered statistically significant.

### Reliability analysis

To evaluate measurement reliability, both intra‐rater and inter‐rater reliability were assessed. For intra‐rater reliability, the same examiner repeated ROI placement and fluorescence intensity measurements after an interval of at least two weeks using identical image processing procedures. Reliability was quantified using the intraclass correlation coefficient based on a two‐way mixed‐effects model for single measurements with absolute agreement ICC(3,1). For inter‐rater reliability, ROI measurements were independently performed by two examiners using the same standardised protocol. Per‐knee mean fluorescence intensity values were calculated from the defined ROIs, and inter‐rater agreement was evaluated using a two‐way random‐effects model ICC(2,1).

All reliability estimates are reported with 95% confidence intervals. All statistical analyses were performed using EZR (Saitama Medical Center, Jichi Medical University, Saitama, Japan), a graphical user interface for R.

All analyses were conducted within the framework of this exploratory feasibility study.

## RESULTS

A total of 32 knees were included (mean age, 38.5 ± 18.1 years; range, 16–66 years; 20 males, 12 females; mean BMI, 25.4 ± 4.4 (Table 1a).

**Table 1:** Patient demographics and surgical procedures.

(a) Patient demographics	
Variable	Value
Age (years), mean ± SD	38.5 ± 18.1
Sex, Male/Female (*n*)	20/12
Body mass index (kg/m²), mean ± SD	25.4 ± 4.4

**(b) Primary surgical procedures**	
**Primary surgical procedure**	**Number of cases**
Meniscal repair	3
Anterior cruciate ligament reconstruction (ACLR)	6
Arthroscopy with hardware removal after ACLR	7
High tibial osteotomy (HTO)	4
Arthroscopy with hardware removal after HTO	6
Posterior cruciate ligament reconstruction (PCLR)	1
Revision ACL reconstruction	4
Medial patellofemoral ligament reconstruction (MPFLR)	1

The procedures included ACL reconstruction in six cases, arthroscopic evaluation performed concomitantly with elective hardware removal after ACL reconstruction in seven cases, posterior cruciate ligament reconstruction in one case, proximal tibial osteotomy in four cases, and hardware removal following osteotomy in six cases. In cases of hardware removal, arthroscopy was performed as part of the same surgical session for clinical indications, not as a routine planned second‐look procedure. Additionally, revision ACL reconstruction was performed in four cases, meniscal repair or meniscectomy in three cases, and medial patellofemoral ligament reconstruction in one case (Table 1b).

Procedures unrelated to the ACL (e.g., PCL reconstruction) were included in the initial consecutive cohort. Knees without ACL injury or prior ACL reconstruction were categorised as ACL‐intact and included in the quantitative analysis.

### ICG perfusion assessment (all knees)

ICG fluorescence was successfully visualised intra‐articularly in all 32 knees, consistently observed in synovial tissue anterior to the popliteus tendon sulcus.

**Figure 4:**
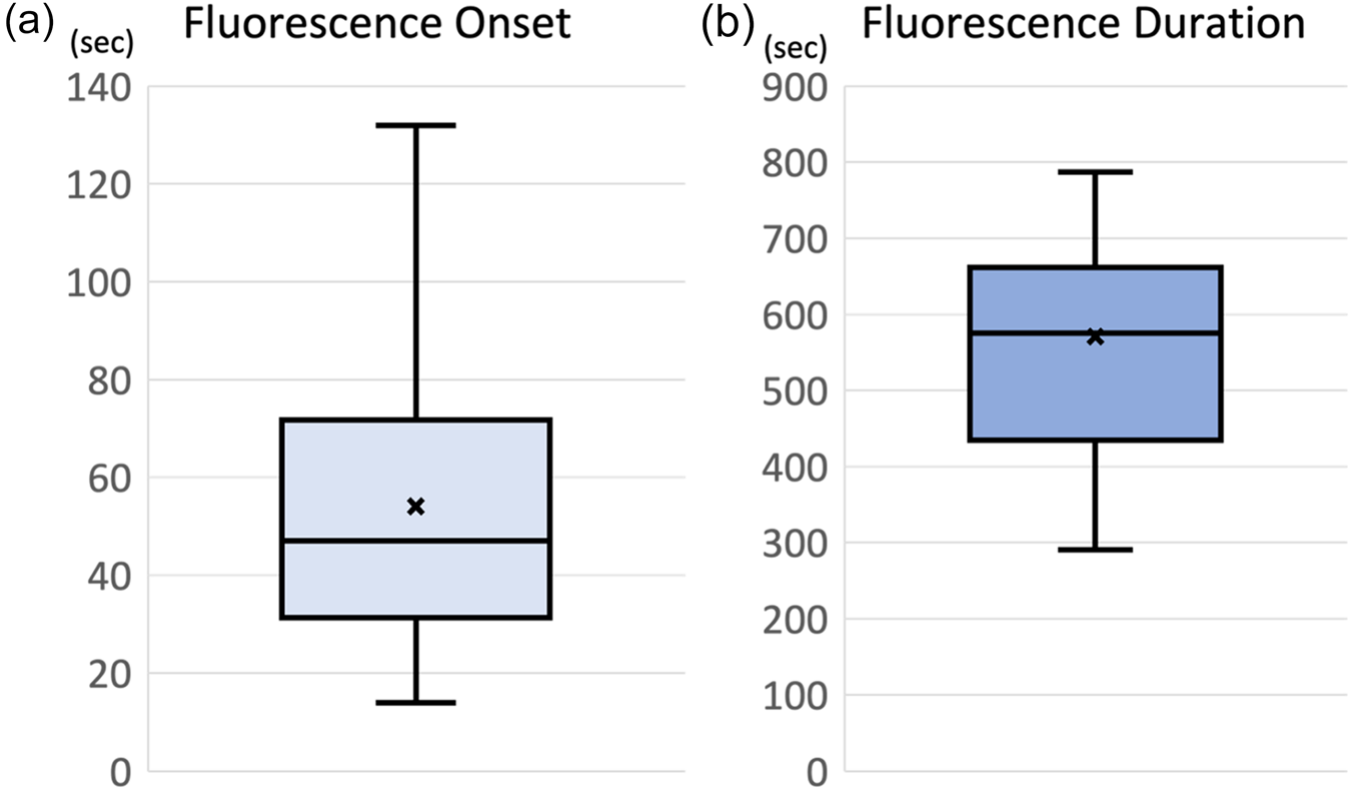
Indocyanine green (ICG) fluorescence onset and duration. ICG fluorescence was consistently observed in all cases. (a) Mean time to fluorescence onset was 53.1 s. (b) Mean fluorescence duration was 577.2 s.

Mean time to fluorescence onset was 53.1 ± 30.7 s, and mean fluorescence duration was 577.2 ± 186.5 s (Figure 4).

No ICG‐related complications were observed.

### ACL rupture cases

In the ACL rupture group (*n* = 6), the mean interval from injury to surgery was 86.9 days (range, 20–210 days), and the mean patient age was 25 years (range, 17–40 years).

**Figure 5:**
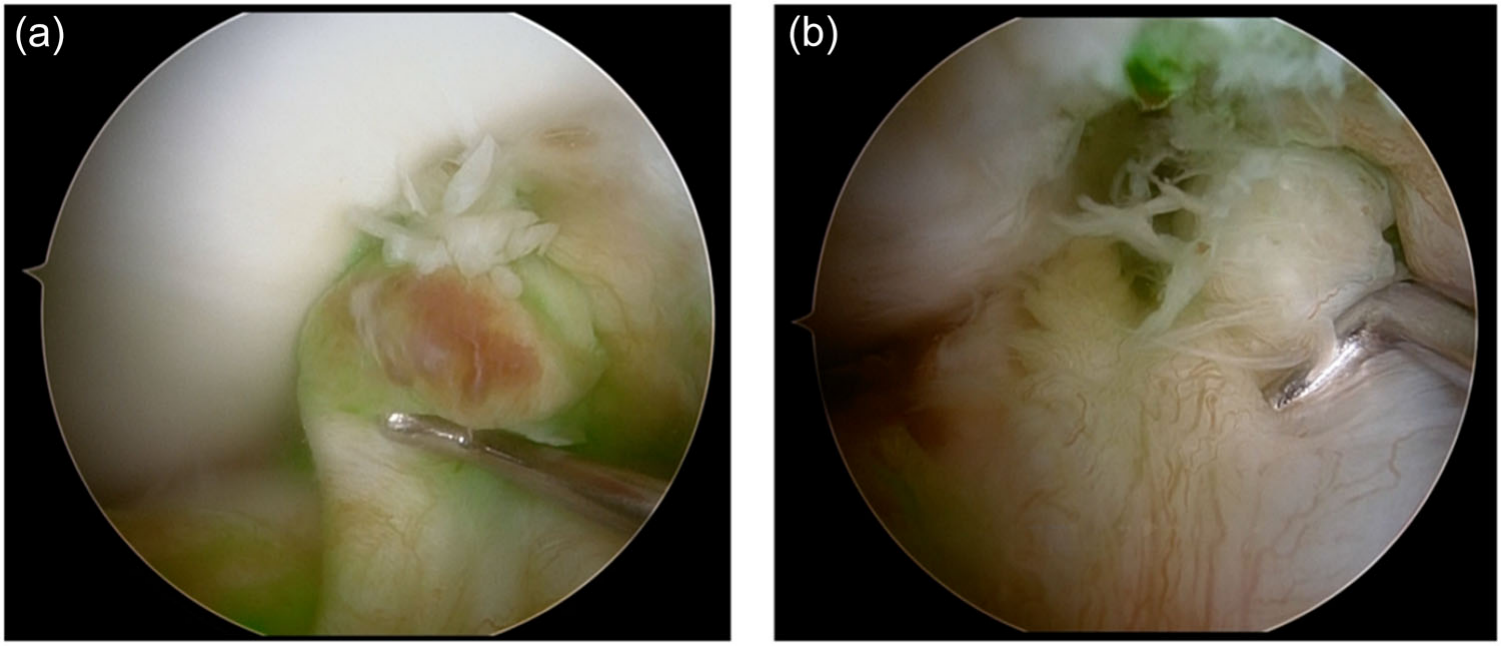
Rupture site of the anterior cruciate ligament (ACL) during primary ACL injury. Representative arthroscopic images demonstrating the complete absence of indocyanine green (ICG) fluorescence at the torn ACL stump in primary ACL rupture. Despite adequate systemic perfusion and clear synovial fluorescence in the surrounding tissues, no fluorescence was observed at the rupture site, indicating a profound local perfusion loss. (a) Right knee. (b) Right knee.

All primary ACL rupture cases demonstrated preserved fluorescence in the surrounding synovium but absence of fluorescence at the torn ACL stump, indicating loss of local perfusion at the rupture site despite preserved intra‐articular perfusion elsewhere in the same knee (Figure 5).

### Quantitative ROI analysis (ACL‐intact knees, *n* = 10)

Per‐knee analysis demonstrated significantly higher fluorescence intensity in the medial portion of the ACL than in the lateral portion (Wilcoxon signed‐rank test, *p* < 0.001).

**Figure 6:**
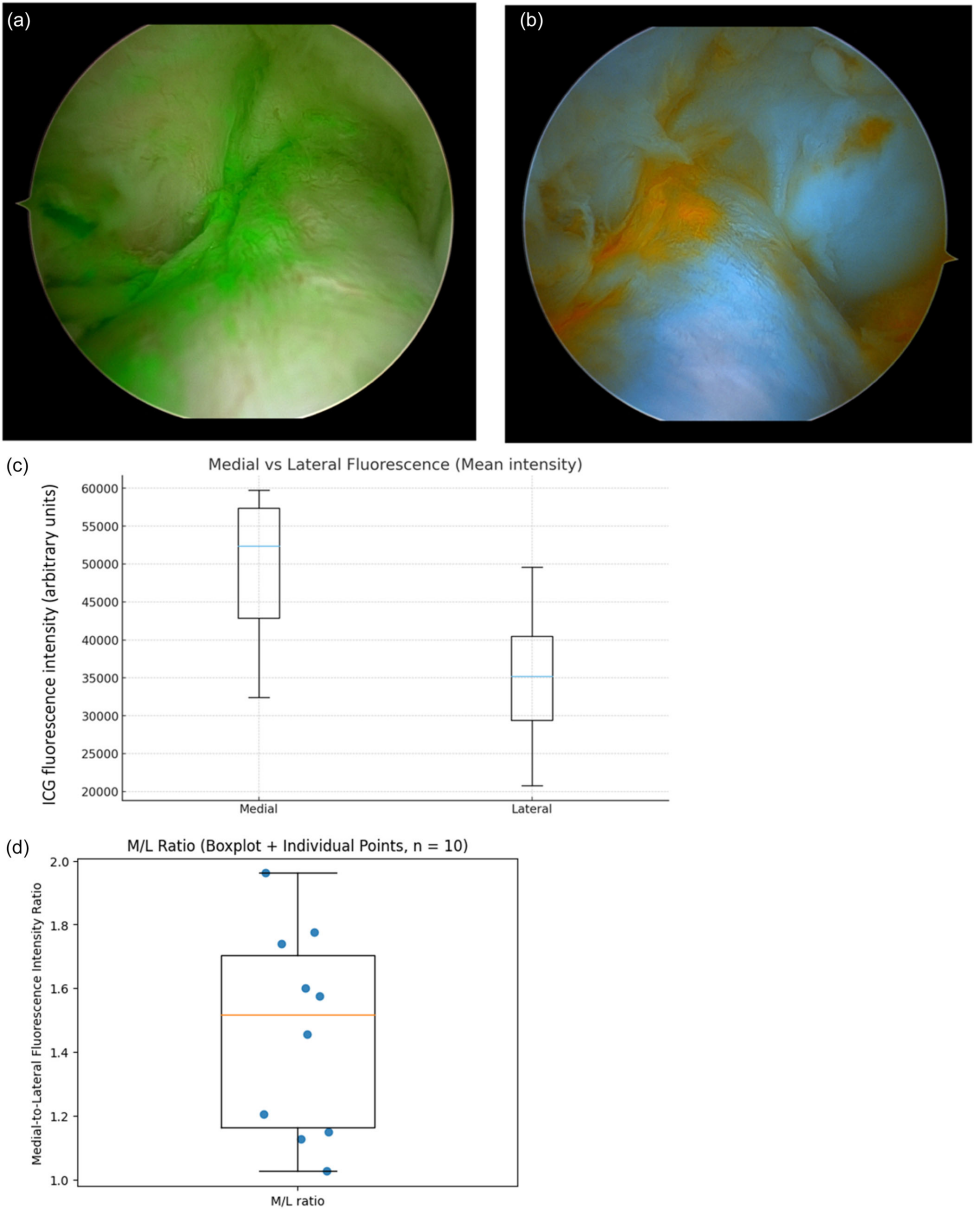
Indocyanine green (ICG) perfusion assessment of intact anterior cruciate ligament (ACL). (a) Representative arthroscopic image of the left knee demonstrating greater fluorescence intensity in the medial portion of the ACL than in the lateral portion of a 50‐year‐old patient (see also Video S1). (b) Corresponding colour‐mapped image illustrating the fluorescence intensity distribution within the ACL. (c) Box‐and‐whisker plots showing per‐knee mean ICG fluorescence intensity in the medial and lateral regions of the ACL in ACL‐intact knees (each value represents the mean derived from three ROIs within the same knee). Boxes represent the interquartile range, horizontal lines indicate the median, and whiskers denote the data range. Box plots display median and interquartile ranges for visualisation purposes. Medial regions demonstrated significantly higher fluorescence intensity than lateral regions (Wilcoxon signed‐rank test, *p* < 0.001). (d) Box‐and‐whisker plot of the medial‐to‐lateral fluorescence intensity ratio (M/L ratio) in ACL‐intact knees (*n* = 10). Boxes indicate the interquartile range, horizontal lines represent the median, and whiskers denote the data range. Individual knees are shown as jittered points.

The distribution of per‐knee medial and lateral fluorescence intensities is illustrated using box‐and‐whisker plots (Figure 6c) to visualise inter‐knee variability.

The M/L ratio exceeded 1.0 in all knees, with a mean ratio of 1.46 (range, 1.03–1.96), indicating consistent medial‐dominant perfusion within the ACL (Figure 6d).

#### Measurement reliability

Intra‐rater reliability of ROI‐based fluorescence intensity measurements was good, with an intraclass correlation coefficient of ICC (3,1) = 0.84 (95% CI, 0.75–0.91).

Inter‐rater reliability based on per‐knee mean fluorescence intensity values demonstrated moderate agreement, with ICC (2,1) = 0.71 (95% CI, 0.34–0.90).

Similar results were obtained using a two‐way mixed‐effects model ICC(3,1), which yielded an ICC of 0.86 (95% CI, 0.67–0.95), indicating good reliability.

These quantitative and qualitative findings are further illustrated by the supplemental videos.

Video S1 demonstrates real‐time perfusion of the synovium anterior to the popliteus tendon sulcus followed by characteristic medial‐dominant perfusion of an intact ACL, whereas Video S2 shows preserved synovial perfusion with complete absence of fluorescence at the ruptured ACL stump.

## DISCUSSION

The most important finding of this study is that ICG perfusion assessment can be safely and reliably performed during routine knee arthroscopy, establishing its feasibility as an intraoperative biological imaging technique. In this consecutive clinical series, ICG fluorescence was successfully visualised in all knees without complications, confirming that real‐time intra‐articular perfusion assessment is technically achievable within standard arthroscopic workflows.

Consistent visualisation of fluorescence, predictable onset timing, and the absence of ICG‐related adverse events indicate that ICG perfusion assessment can be integrated into standard arthroscopic procedures without additional risk or procedural complexity. Importantly, the observed fluorescence duration of approximately 10 min provides a clinically meaningful working window, allowing comprehensive biological assessment without the need for repeated injections. These characteristics support the practicality of this technique as an intraoperative tool rather than an experimental adjunct and were visually corroborated by the supplemental videos demonstrating synovial perfusion followed by medial‐dominant fluorescence in intact ACLs, contrasted with complete absence of fluorescence at rupture sites. The present study provides the first clinical description of intra‐articular ICG perfusion assessment of the ACL during routine knee arthroscopy.

Beyond feasibility, this study demonstrated characteristic perfusion patterns within the ACL. In ACL‐intact knees, fluorescence intensity was consistently greater in the medial portion of the ligament, a distribution that aligns with established descriptions of the ACL vascular supply, including contributions from the middle genicular artery [2, 5, 31]. This reproducible medial‐dominant pattern supports the biological validity of intra‐articular perfusion visualisation. Conversely, all primary ACL rupture cases demonstrated a complete absence of fluorescence at the torn stump, indicating profound local perfusion loss [17, 18, 24]. Importantly, the primary conclusions of the present study are derived from within‐knee comparisons of regional fluorescence intensity rather than comparisons between patients or surgical indications. This design reduces the potential confounding effects related to heterogeneous surgical backgrounds.

From a clinical perspective, these findings suggest that real‐time perfusion assessment may help identify biologically viable ACL tissue during arthroscopy. Such biological information is not available through conventional structural visualisation alone. In line with recent interest in biologically oriented ACL preservation strategies, including primary repair techniques, intraoperative perfusion assessment may be particularly relevant in procedures such as ACL repair or in cases of partial ACL injury, where tissue viability may influence surgical strategy [7, 12, 21, 26]. Although partial‐thickness tears were not included in this cohort, extending perfusion assessment to such injuries may be informative for biologically driven decision‐making. Given the exploratory nature of the present study, these implications should be interpreted as hypothesis‐generating.

Taken together, these observations extend previous reports of ICG fluorescence imaging in other surgical fields and limited orthopaedic applications by demonstrating that quantitative, real‐time assessment of intra‐articular microcirculation is feasible during routine knee arthroscopy.

Previous orthopaedic reports have demonstrated the feasibility of ICG fluorescence imaging during arthroscopy, including applications in the shoulder joint and intra‐articular evaluation of meniscal tissue [14, 29]. However, these studies focused on different anatomical structures and did not specifically examine perfusion characteristics of the ACL or regional perfusion differences within the ligament substance. In the present study, absence of fluorescence at ruptured ACL stumps was observed despite preserved synovial perfusion, and a consistent medial‐dominant perfusion pattern was identified in intact ACLs (medial > lateral). This regional distribution may reflect underlying vascular anatomy and provides additional context for interpreting perfusion loss in rupture cases. Together, these findings extend prior intra‐articular applications of ICG fluorescence by characterising ACL‐specific perfusion patterns in a clinical arthroscopic setting.

## BIOLOGICAL CONTRIBUTION TO ARTHROSCOPY

Despite substantial advances in arthroscopic visualisation, previous techniques have focused almost exclusively on structural assessments. ICG perfusion assessment introduces a biological dimension by enabling direct visualisation of intra‐articular microcirculation in vivo. By bridging the gap between morphological inspection and tissue viability, this approach may become increasingly relevant in biologically sensitive procedures such as ligament repair, meniscal preservation, and cartilage restoration. Moreover, the quantitative nature of perfusion data—including onset, duration, and intensity mapping, is compatible with future AI and machine learning frameworks [6, 10]. Although manual ROI definition remains a limitation, automated computer vision–based vascular mapping may further enable real‐time objective biological assessment during surgery [27].

### Prospective value

Although postoperative healing and clinical outcomes were not assessed, our findings provide a necessary biological foundation for future outcome‐oriented investigations. Establishing the feasibility and reproducibility of intra‐articular perfusion visualisation is an essential first step before perfusion‐guided treatment strategies and outcome predictions can be meaningfully explored. Future studies should include larger prospective cohorts, standardised acquisition and quantification protocols, within‐patient comparisons when feasible, and longitudinal outcome correlations to determine whether perfusion patterns can be translated into validated intraoperative decision frameworks.

### Clinical relevance

From a practical perspective, intraoperative ICG perfusion assessment may provide surgeons with supplementary biological insight during routine arthroscopy without altering standard workflow. Visualisation of regional perfusion patterns may assist in distinguishing viable ligament tissue from hypovascular rupture stumps and may complement conventional structural assessment during arthroscopy. While treatment thresholds cannot be defined based on the present data, this technique may serve as a feasible adjunct in day‐to‐day arthroscopic practice.

### Limitations

This study has a few limitations. First, the sample size was modest and the cohort was heterogeneous with respect to surgical indications. Larger studies are required to confirm reproducibility across broader clinical settings and to better characterise inter‐individual variability in intra‐articular vascularity. Second, although ROI‐based quantification was performed, standardised methods for arthroscopic ICG signal measurement remain incompletely established. Fluorescence intensity may be influenced by technical factors such as camera distance, illumination angle, irrigation conditions, and image compression; therefore, a ratio‐based approach was adopted to mitigate acquisition‐related variability. Third, a direct within‐knee comparison between normal and injured tissues was not possible, limiting our ability to distinguish intrinsic regional vascularity from pathology‐related changes at the individual level. Fourth, postoperative healing and clinical outcomes were not assessed; therefore, the prognostic value of the intraoperative perfusion findings remains undetermined. Finally, perfusion‐guided treatment algorithms and actionable thresholds for surgical decision‐making were not defined in this study.

## CONCLUSION

ICG perfusion assessment adds a biologically oriented perspective to knee arthroscopy by enabling safe, real‐time visualisation of intra‐articular vascularity. In this study, the technique demonstrated reproducible ACL perfusion patterns in intact ligaments and consistently identified absence of perfusion at ACL rupture sites, providing intraoperative insight into tissue vascularity.

Although further validation is required, intra‐articular perfusion assessment may serve as a feasible adjunct to conventional structural evaluation during arthroscopy. By offering real‐time information on regional tissue perfusion, this approach has the potential to support biologically informed intraoperative decision‐making in selected procedures. In addition, the quantitative framework for intra‐articular ICG perfusion assessment described here may facilitate future development of standardised perfusion metrics and integration with advanced image analysis systems.

## AUTHOR CONTRIBUTIONS

Yasutoshi Ikeda contributed to the study conceptualisation and design, methodology, data acquisition, data curation, formal analysis and drafting of the manuscript. Kazushi Horita and Kodai Hamaoka contributed to data acquisition and data curation. Shinichiro Okimura contributed to data acquisition and methodology. Yohei Okada contributed to data acquisition. Tomoaki Kamiya contributed to methodology and supervision. Makoto Emori contributed to formal analysis and visualisation. Atsushi Teramoto contributed to study conceptualisation, supervision and project administration. All authors critically revised the manuscript and approved the final version.

## CONFLICT OF INTEREST STATEMENT

The authors declare no conflicts of interest.

## ETHICS STATEMENT

This prospective clinical study was approved by the Institutional Review Board of Sapporo Medical University (approval date: 23 May 2025; approval number: 170–20) and was conducted in accordance with the Declaration of Helsinki. Written informed consent was obtained from all participants prior to inclusion in the study.

## Supporting information


**Supplementary Video 1** Real‐time indocyanine green (ICG) perfusion assessment during knee arthroscopy demonstrated fluorescence onset in the synovium anterior to the popliteus tendon sulcus approximately 50 s after intravenous injection, followed by characteristic medial‐dominant perfusion of the intact anterior cruciate ligament (ACL).

**Supplementary Video 2** Real‐time indocyanine green (ICG) perfusion assessment during knee arthroscopy demonstrated the absence of fluorescence at the torn anterior cruciate ligament (ACL) stump despite adequate synovial enhancement, indicating a complete loss of local perfusion at the rupture site.

## Data Availability

The data supporting the findings of this study are available from the corresponding author upon reasonable request.
